# Rapid and quantitative detection of DNA hybridization using a simplified Fabry–Perot interferometric biosensor

**DOI:** 10.1039/d4ra01390e

**Published:** 2024-04-24

**Authors:** Xin Shi, Yanhong Ma, Yipeng Liao, Hoi Lut Ho

**Affiliations:** a School of Microelectronics, Shenzhen Institute of Information Technology Shenzhen China shix@sziit.edu.cn; b Department of Electrical and Electronic Engineering, The Hong Kong Polytechnic University Kowloon Hong Kong China

## Abstract

This study introduces a miniaturized fiber-optic Fabry–Perot (FP) interferometric biosensor, distinctively engineered for cost-effective, rapid, and quantitative DNA sequence detection. By leveraging the interference patterns generated within a Fabry–Perot microcavity, our sensor precisely monitors DNA hybridization events in real-time. We have verified the sensor's biofunctionalization *via* fluorescent labeling and have extensively validated its performance through numerous hybridization and regeneration cycles with 1 μM single-stranded DNA (ssDNA) solutions. Demonstrating remarkable repeatability and reusability, the sensor effectively discerns ssDNA sequences exhibiting varying degrees of mismatches. Its ability to accurately distinguish between sequences with 2 and 7 mismatches underscores its potential as a valuable asset for swift DNA analysis. Characterized by its rapid response time—typically yielding results within 6 minutes—and its adeptness at mismatch identification, our biosensor stands as a potent tool for facilitating accelerated DNA diagnostics and research.

## Abstract 2

The simple-structured Fabry–Perot interferometric biosensor is capable of rapid and quantitative detection of DNA hybridization.

## Graphical abstract

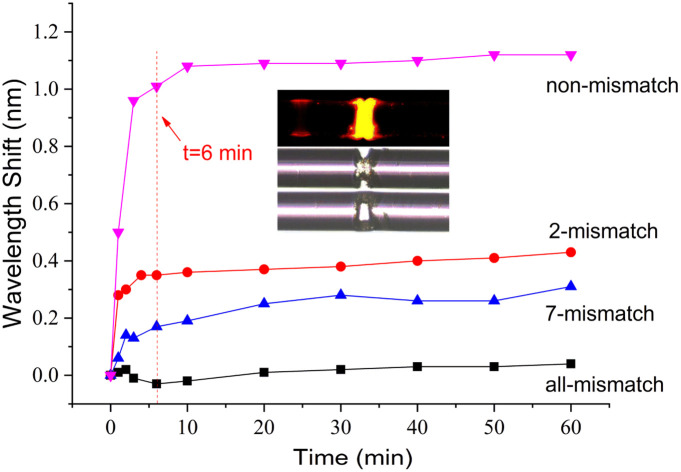

## 1. Introduction

As biotechnology progresses, the pivotal role of biosensors in domains such as public health, environmental sciences, biological engineering, disease diagnostics, and pharmaceutical research has become increasingly evident.^1–3^ Currently, a diverse array of biosensing methods is utilized, including optical, electrochemical, fluorescent, thermometric, and magnetic techniques.^4–8^ Among these, fiber-optic biosensors stand out for their compactness, chemical inertness, superior biocompatibility, resistance to electromagnetic interference, and notably, their capacity for label-free and real-time detection.^9,10^ These attributes have contributed to their rapid development in recent years.

Innovative designs, such as fiber Bragg gratings,^11–13^ long period fiber gratings,^14–17^ fiber interferometers,^18–20^ surface plasmon resonance,^21–24^ lossy mode resonance,^25^ and specialty fiber couplers,^26^ have been pivotal in advancing fiber-optic biosensors. These sensors typically detect biological phenomena by measuring changes in the refractive index (RI) of an analyte or the thickness of a biofilm, modulating optical parameters for sensitive and label-free detection.

Focusing on DNA hybridization detection, significant emphasis has been placed on microfibers,^27^ grating technologies,^28^ and integrated strategies.^29^ Techniques like tapering or etching reduce optical fiber diameters, enhancing light–material interactions for high RI sensitivity. However, these advanced sensors often face challenges related to their delicate structure and packaging difficulties. Furthermore, achieving high sensitivity through multiple interference effects, such as the Vernier effect,^30^ introduces complexities in signal demodulation. In DNA biological solutions, the pronounced sensitivity to RI changes necessitates temperature control measures to ensure accuracy.

This paper addresses key challenges in fiber-optic biosensor development, focusing on packaging complexities and temperature stability. We introduce a miniaturized fiber-optic Fabry–Perot biosensor that simplifies the sensor's design and signal interpretation system, significantly reducing costs while maintaining high efficacy. Through empirical studies, including biofunctionalization validation *via* fluorescent labeling and repeated ssDNA solution measurements with varying mismatches, we demonstrate the sensor's reusability, reliability, and practical applicability. Our findings underscore the fiber-optic Fabry–Perot biosensor as a substantial breakthrough, offering a cost-efficient and manufacturable solution to prevailing biosensing challenges.

## 2. Experimental

### 2.1 Sensor design and fabrication

**Fig. 1:**
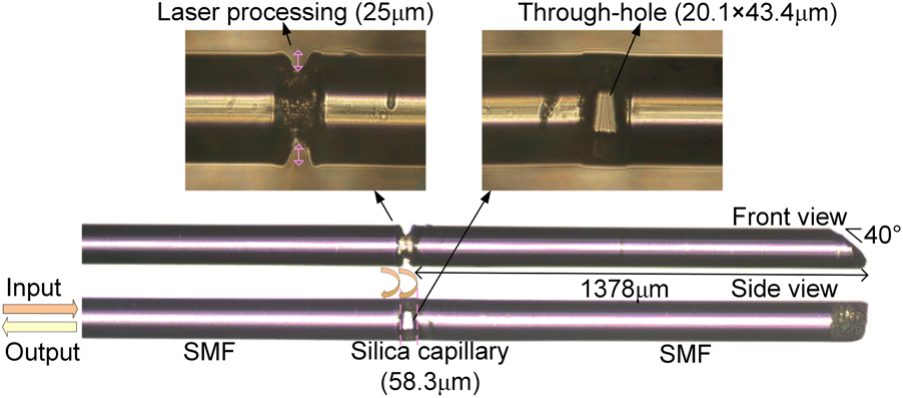
The structure of fiber-optic Fabry–Perot interferometric sensor.

We designed and fabricated an intracavity fiber-optic Fabry–Perot sensor, depicted in Fig. 1. The sensor's structure consists of three segments: two common single-mode fibers (SMF) and an interposed silica capillary tube. With a 75 μm inner diameter, the silica capillary complements the single-mode fiber (SMF), as both possess a uniform outer diameter of 125 μm. This uniformity streamlines the cleaving and splicing operations, making it possible to employ standard fiber cleaving and fusion splicing tools.

The construction of the sensor begins with the fusion of a SMF to a silica capillary tube using an automated fiber fusion splicer, ensuring consistent and reliable welding. The next step involves trimming the assembly with a fiber cleaver to leave approximately a 58.3 μm segment of the capillary tube, which is then fused to another segment of SMF, completing the sensor structure.

The capillary tube's trimming, which aims to maintain a minimal and precise length, achieves a notably high success rate, indirectly confirming the robustness of the fiber's welding to the capillary tube. The cutting stage, critical for precision, requires the fiber cleaver to be mounted on an optical adjustment stand, allowing for fine control. The accuracy of this step is enhanced by visual guidance from a CCD camera, providing an enlarged image for meticulous alignment and execution.

An increase in the inner diameter of capillaries inversely affects wall thickness, necessitating a specific wall thickness for the successful fusion of SMFs and capillaries to maintain structural integrity. The length of the capillary, or more precisely, the cavity length of the FP interferometer, exerts a more significant influence on sensor functionality than its inner diameter. In aqueous environments, a reduction in the FP microcavity length leads to enhanced RI sensitivity, while simultaneously decreasing temperature sensitivity. This phenomenon underscores the critical balance between structural dimensions and the optical properties essential for optimizing sensor performance in liquid mediums.

To construct the biosensor's microcavity, femtosecond laser processing was utilized to intricately etch two small gaps at both the upper and lower ends of the cavity, each with a laser processing depth of 25 μm. This technique carved out a through-hole measuring 20.1 × 43.4 μm, as can be seen in the side view, which facilitates the smooth ingress and egress of fluids within the cavity.

While a singular micro-hole might permit the entry of liquids, it also poses a risk of bubble entrapment, which could severely disrupt sensor performance. Nevertheless, the introduction of micro-holes does present a trade-off with the mechanical strength of the sensors. Thus, forthcoming research endeavours will be directed towards achieving an optimal equilibrium: minimal invasive processing that upholds efficient fluid dynamics without compromising the sensor's structural integrity.

In the meticulous design of the intracavity fiber-optic Fabry–Perot sensor, careful consideration is given to its physical configuration to optimize measurement accuracy and sensor performance. One end of the sensor is seamlessly integrated with an optical measurement system, while the opposite end is deliberately left with several millimeters reserved with angled end face. Such angling can be achieved through the use of a femtosecond laser, or alternatively, through manual adjustments using pen-type optic-fiber cutting knives for practical ease.

As illustrated in Fig. 1, the measured distance from the FP cavity to the obliquely cut end approximates 1378 μm. This design ensures that the length of the fiber extending from the microcavity to the beveled end face significantly exceeds ten times the length of the cavity itself. This design consideration is crucial for reducing the influence of reflections from the beveled end face on the sensor's internal reflective surfaces. By significantly diminishing these extraneous reflections, the design facilitates the simplification of spectral signal demodulation.

While the femtosecond laser processing employed in our experiments is associated with high equipment costs, potentially elevating the manufacturing expense of the sensor, it's important to note that there are alternative, more cost-effective methods available. Techniques such as grinding, polishing, and chemical etching can be utilized to reduce production costs. In our experiments, the primary objective was to leverage the existing equipment to demonstrate the feasibility of the technology. Our focus was on validating the concept and establishing a proof of principle, rather than on optimizing the cost-efficiency of the manufacturing process at this stage.

The operation of the fiber-optic FP sensor, as illustrated in Fig. 1, is based on the principle of dual-beam interference within the FP cavity. This mechanism allows for the precise measurement of light intensity variations due to interference effects, which can be represented as:1
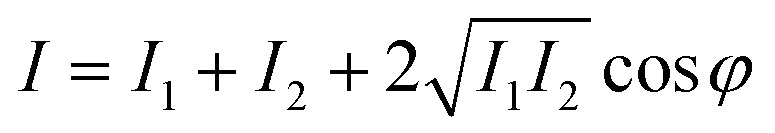
where *I* denotes the total intensity of the interference light, *I*_1_ and *I*_2_ represent the intensities of light at the two reflective surfaces within the FP cavity, and *ϕ* is the phase difference induced by the cavity's optical path difference.

Furthermore, the wavelength at which destructive interference leads to the formation of spectral troughs, or minimum intensity points, is given by:2
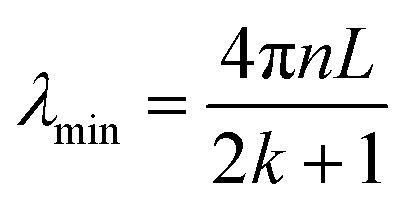
In this expression, *n* stands for the RI of the medium within the cavity, *k* represents the wave vector, and *L* denotes the cavity length.

As indicated by eqn (2), the spectral shift of the troughs is dependent upon the RI within the microcavity and its length. Optical fiber biosensors are characterized by their exceptionally high sensitivity to RI changes. However, the RI of biological solutions is notably susceptible to environmental temperature variations. Consequently, the temperature dependence of optical fiber biosensors within biological solutions emerges as a critical factor that warrants careful consideration.

### 2.2 Experimental setup and optical configuration

**Fig. 2:**
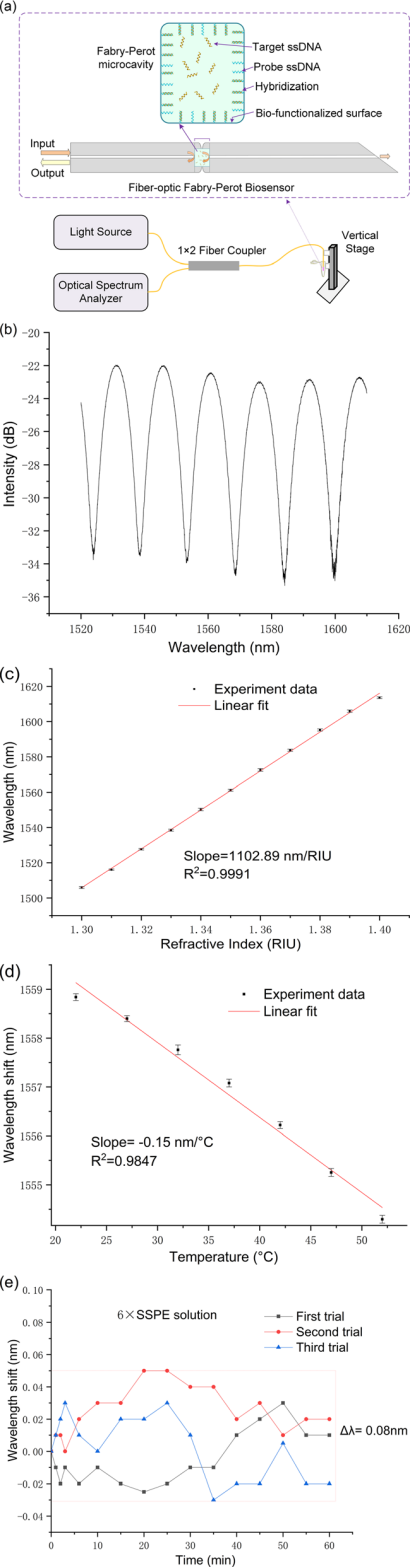
(a) Schematic of the experimental setup for testing the fiber-optic FP biosensor; (b) reflection spectrum of the sensor when immersed in water; (c) RI response of the sensor; (d) temperature response in water withing a heating chamber and (e) conduct three measurements of the sensor's temperature stability at ambient room temperature (25 °C) within a 6× SSPE buffer solution. The error bars are calculated from the standard deviation of three repeated measurements.

The fiber-optic Fabry–Perot sensor, being a reflective sensor, was characterized using the experimental configuration depicted in Fig. 2a. Illumination is provided by a broadband light source (ASE, a wavelength range of 1520–1610 nm, an output power of 50 mW), with the emitted light directed through a 1 × 2 fiber-optic coupler towards the FP microcavity. This cavity, upon immersion in a target ssDNA biological solution, exhibits variations in the optical signal in response to DNA hybridization occurring within the microcavity. These changes are reflected through the same coupler and captured by the optical spectrum analyzer (OSA: AQ6370C).

To achieve precise control over the sensor probe's immersion time in the test solution, a vertical stage was utilized, facilitating accurate, time-correlated data collection. It is crucial to note that during each measurement cycle, the commencement of data recording was deliberately delayed until 20 seconds after the sensor's immersion in the target ssDNA solution. This delay is strategically implemented to account for transient fluctuations that typically occur immediately after the sensor is introduced to the solution. Such fluctuations can significantly impact the initial measurements, thereby necessitating a short waiting period to allow the system to stabilize.

Fig. 2b displays the reflection spectrum of the fiber-optic Fabry–Perot sensor when submerged in water, showcasing a prominent interference pattern predominantly resulting from the reflections at the two end faces of the sensor's hollow micro-cavities. Within the wavelength range of 1520 to 1610 nm, numerous peaks and troughs are discernible, with the specific wavelengths of these troughs being influenced by both the refractive index of the solution within the cavity and the cavity's length. Notably, slight variations in the refractive index, such as those caused by DNA hybridization, lead to shifts in these trough wavelengths.

For the detection process, any one of these troughs can be utilized, provided it aligns with the operational wavelength of both the light source and the detector within the measurement system. By selecting a wavelength that falls within the operational range of standard optical communication devices, the sensor facilitates straightforward integration with cost-effective optical components, such as couplers, semiconductor laser sources, and detectors. This strategic compatibility greatly enhances the sensor's utility by allowing it to leverage existing optical infrastructures, thus boosting its efficiency and significantly lowering the costs associated with its implementation.

Fig. 2c illustrates the refractive index (RI) sensitivity of the fiber-optic Fabry–Perot sensor, showcasing how the sensor's spectral shifts correspond to solutions with varying refractive indices. The sensor's refractive index sensitivity is determined to be 1102.89 nm per RIU, placing it within the moderate sensitivity range. This level of sensitivity is indicative of the sensor's potential capability to detect subtle changes in the refractive index of surrounding mediums, which is essential for accurate biosensing applications.

Fig. 2d delves into the temperature responsiveness of our sensor, showcasing its behavior under varying thermal conditions. Through precise measurements conducted within a controlled heating chamber, the sensor demonstrated a temperature sensitivity of −0.15 nm °C^−1^. This relatively low temperature sensitivity indicates the sensor's robust performance in environments with temperature fluctuations, underscoring its capability for reliable DNA detection even at ambient room temperatures.

6× Saline-Sodium Phosphate-EDTA (SSPE) play pivotal roles in DNA hybridization stage. The stability of the developed sensor was evaluated in 6× SSPE solution (without target ssDNA) at room temperature over a duration of 60 minutes. The minimal observed wavelength shift of 0.08 nm as indicated in Fig. 2e, staying well under the 0.1 nm threshold, signifies the sensor's high stability in DNA biological solutions.

Collectively, the data presented in Fig. 2c–e highlight the fiber-optic FP sensor's robust performance under a variety of test conditions. Its demonstrated moderate sensitivity to refractive index changes, relatively low temperature sensitivity in biological solutions illustrate the sensor's potential for effective application in biosensing, particularly in the precise detection of DNA hybridization events.

### 2.3 Chemical and materials

The following reagents and materials were used: (3-aminopropyl)triethoxysilane (APTES), sodium dodecyl sulfate (SDS) and phosphate-buffered saline (PBS) were acquired from Sigma-Aldrich (United Kingdom). Dimethyl suberimidate (DMS), hydrochloric acid (HCl), and methanol were procured from Thermo Fisher Scientific Inc. Single-stranded DNA (ssDNA) with varying base sequences (detailed in Table 1) and fluorescently labeled with TAMRA, were sourced from Shanghai Medicilon Inc. (Shanghai, China). The 6× SSPE buffer, which we prepare in our laboratory, consists of a solution with 0.9 M NaCl, 0.06 M NaH_2_PO_4_, and 0.006 M EDTA.

**Table 1:** Sequences and modifications of probe and target oligonucleotides

Oligonucleotide	5′ end	Sequence (5′ to 3′)	3′ end
Probe	None	GCACAGTCAGTCGCC	NH_2_
Target (perfectly match)	None	GGCGACTGACTGTGC	None
Target (fluorescent label)	None	GGCGACTGACTGTGC	TAMRA
Completely mismatch	None	TTATCAGTCAGTGTA	None
2 mismatches	None	GTCGACTTACTGTGC	None
7 mismatches	None	TTAGACGTACTTTGA	None

Table 1 presents the sequences of DNA used in our study, including an oligonucleotide labeled with TAMRA (tetramethylrhodamine), which serves to confirm the efficacy of our biofunctionalization process. After Successful detection of target ssDNA was achieved through spectral shift analysis, following experiments will extend this approach to assess the sensor's sensitivity to various degrees of mismatched ssDNA sequences, such as those with 2, 7, and complete mismatches, as delineated in Table 1. Sequences with complete mismatches will also provide a crucial negative control, ensuring the validity and reliability of our experimental results.

### 2.4 Surface functionalization

**Fig. 3:**
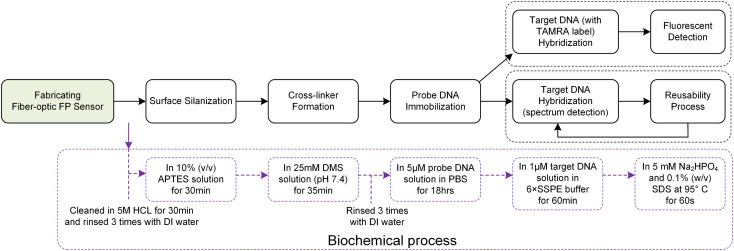
Schematic representation of the biofunctionalization process and hybridization detection.

The biochemical processing of the sensor surface is meticulously detailed in Fig. 3, highlighting the specific reagent concentrations and reaction times utilized at each stage. The process initiates with the surface modification of the sensor through silanization, where hydroxyl groups on the sensor's surfaces react with APTES to introduce primary amine groups. This step is crucial for facilitating subsequent chemical reactions. Following a thorough cleaning with deionized (DI) water, the sensor is then immersed in a DMS solution, forming imidoester-terminated linkers on its surface. These linkers are primed for covalent bonding with primary amines present in proteins or N-terminated oligonucleotides.^31,32^

Probe DNA immobilization is achieved by incubating the sensor in a 5 μM probe ssDNA solution in PBS for 18 hours. After immobilization, the sensor is cleaned with DI water, followed by a rinse in 6× SSPE buffer, and incubated in a 1 μM target DNA solution in 6× SSPE for 60 minutes. Quality control measures, including fluorescence labeling, are employed to assess the attachment's density and uniformity. This rigorous process ensures the sensor's high efficacy for precise DNA hybridization detection.

Fig. 3 presents a comparative analysis of two methodologies for detecting DNA hybridization: fluorescence detection and spectral analysis. Fluorescence detection employs TAMRA-labeled target ssDNA, enabling the visualization of hybridization events. In contrast, spectral analysis offers the advantage of *in situ* and real-time monitoring, providing immediate insights into the hybridization process. Despite their operational differences, both methodologies share a core procedural framework that includes essential steps such as surface preparation, probe attachment, and the facilitation of target hybridization.

Notably, except for the probe ssDNA immobilization phase, which requires up to 18 hours, all other procedural stages—including the crucial step of hybridization verification—are completed within a concise 60 minute timeframe. To maintain surface integrity and mitigate the risk of cross-contamination, each phase is interspersed with rigorous cleansing DI water.

## 3. Results and discussion

### 3.1 Fluorescence validation

In our study, we utilized fluorescence detection to ascertain the effectiveness of the sensor's surface biofunctionalization. The experimental procedure involved immersing both a probe-loaded fiber and a control fiber in a solution containing 1 μM of perfectly matched target ssDNA for a duration of 60 minutes to facilitate hybridization.

**Fig. 4:**
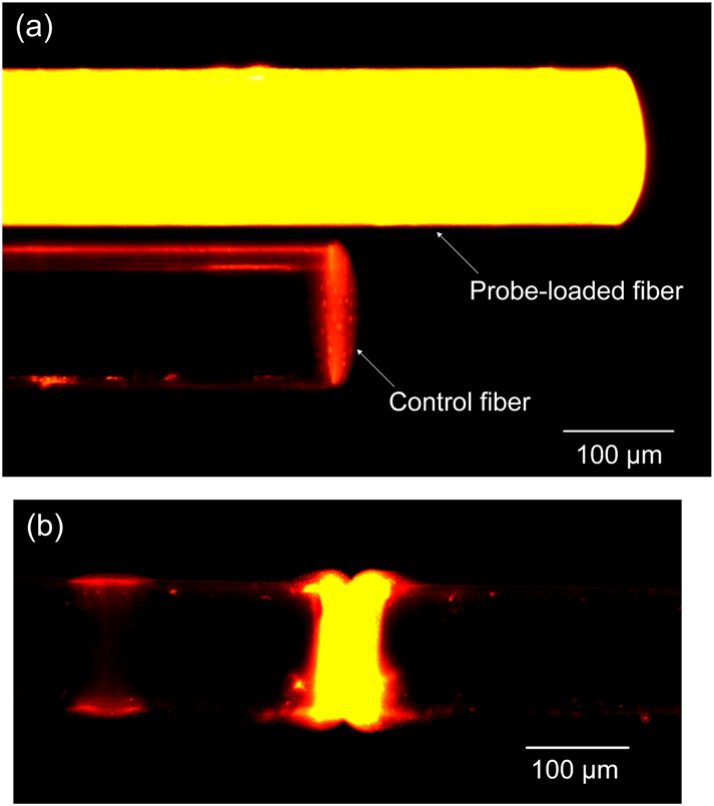
(a) Comparative hybridization results of probe-loaded (processed) and control (unprocessed) fibers and (b) fluorescence imaging of the fiber-optic Fabry–Perot cavity.

Post-hybridization, the samples were subjected to analysis under a fluorescence microscope. The analysis utilized a 553 nm green light for excitation, with the observation of a 578 nm yellow fluorescence indicating successful hybridization. This yellow fluorescence was discerned after the green excitation light was filtered out, ensuring that only the relevant fluorescence signals were captured. Notably, as depicted in Fig. 4a, the probe-loaded fiber displayed a significantly stronger emission of yellow fluorescence compared to the control sample. The control samples, which lacked the probe DNA served as a reference point for establishing the baseline level of fluorescence background noise. The pronounced disparity in fluorescence intensity between the probe-loaded fiber and the control vividly demonstrates the successful immobilization and hybridization of the target ssDNA onto the probe-loaded fiber.

Further investigation into the fluorescence outcomes within the Fabry–Perot microcavity, as shown in Fig. 4b, revealed bright and uniform yellow fluorescence signals across the microcavity. This uniformity and intensity of fluorescence within the microcavity are indicative of successful DNA hybridization, affirming the efficacy of the surface treatment carried out during the biofunctionalization phase.

### 3.2 DNA detection

The fiber-optic Fabry–Perot sensor's capability to detect specific ssDNA sequences through hybridization was rigorously tested. After the immobilization phase, the sensor head was carefully immersed in a 1 μM solution of perfectly matched target ssDNA, using a 6× SSPE buffer as the medium. Over the course of 60 minutes at room temperature, resonance wavelength shifts indicative of hybridization were continuously monitored using an optical spectrum analyzer.

After this initial test, the sensor head was subjected to a regeneration process. This involved immersing it in a stripping buffer (SDS solution) heated to 95 °C for 60 s and then rinsing it with deionized water, preparing it for subsequent tests.

**Fig. 5:**
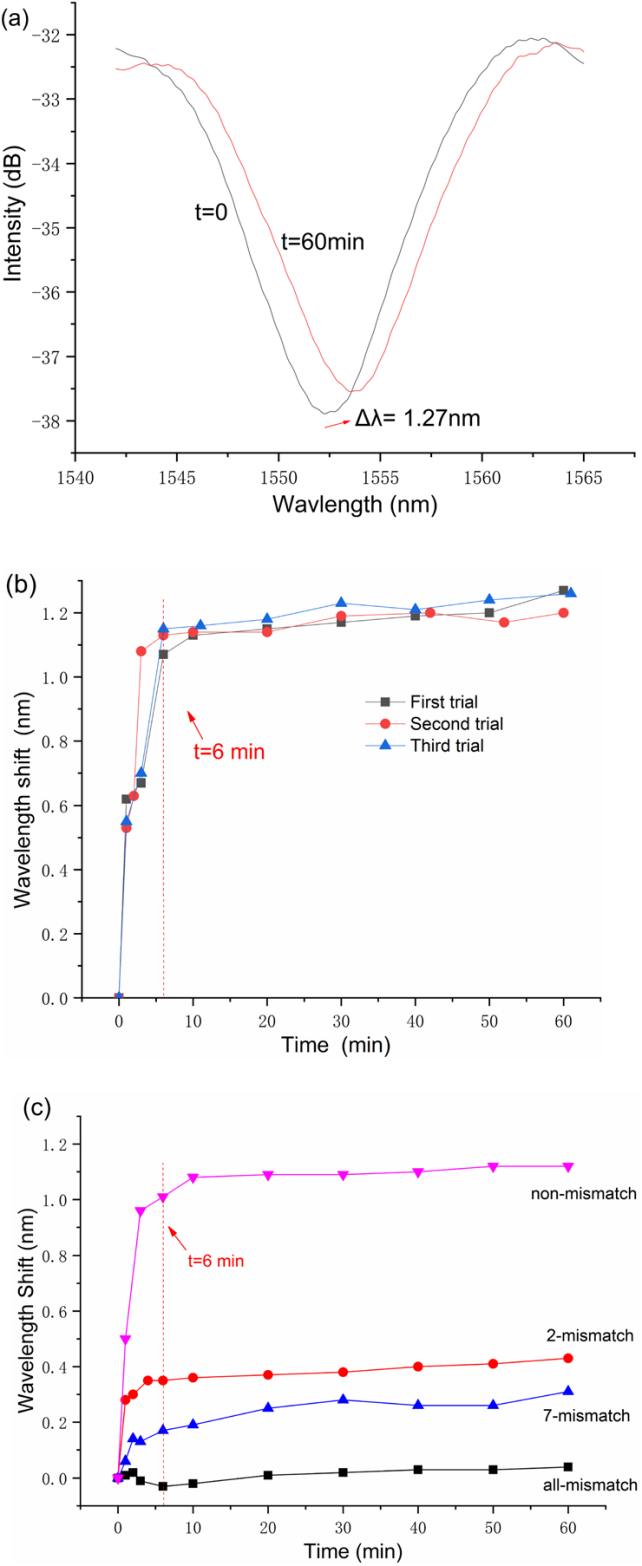
(a) Reflection spectrum of the first trial; (b) comparison of three trials, demonstrating the sensor's repeatability and stability and (c) time-resolved resonance wavelength shifts for the sensor in ssDNA solutions with different mismatch levels.

The reflection spectrum obtained from the initial DNA hybridization measurement reveals a trough drift of 1.27 nm over a 60 minute period, as shown in Fig. 5a. Additionally, the process of demodulating the spectral signal is demonstrated to be straightforward.

Before proceeding with the second and third rounds of trials, the sensor was processed using the same reusable steps. The results of these three separate measurements are collectively presented in Fig. 5b. It is important to note that in each measurement cycle, data recording was initiated 20 seconds after the sensor's immersion in the target ssDNA solution.

The data presented in Fig. 5b illustrate the sensor's dynamic response to target ssDNA, where the resonance wavelength shifts rapidly reach an equilibrium point, surpassing 1 nm within a brief 6 minute window for all three conducted measurements. Notably, beyond this rapid initial adjustment, minimal changes in the resonance wavelength were observed over the duration of an hour. This pattern of response not only demonstrates the sensor's swift adaptability to the presence of target ssDNA but also its stable performance through repeated cycles of use.

After investigation into the repeatability and reusability of the fiber-optic FP biosensor, significant attention was dedicated to its proficiency in discriminating between mismatches in ssDNA sequences during hybridization processes. To this end, a series of 1 μM target ssDNA solutions were meticulously prepared, encompassing sequence similarities—ranging from complete mismatches to perfectly matched sequences, as meticulously catalogued in Table 1. Utilizing the same fiber-optic FP biosensor for four distinct assays, each iteration was succeeded by a regeneration phase, entailing an SDS solution bath at 95 °C for 60 seconds. The assays were methodically conducted in a sequence that progressed from the highest level of mismatches to sequences with no mismatches.

The empirical results, depicted in Fig. 5c, affirm the biosensor's discerning capability to differentiate among varying degrees of ssDNA sequence mismatches. Notably, the sensor efficiently detected the primary hybridization-induced wavelength shifts predominantly within the initial 6–10 minutes of each assay, with minimal fluctuations observed in the rest 50 minutes. This rapid detection capability markedly distinguishes the fiber-optic Fabry–Perot biosensor from other designs, showcasing its suitability for swift and pragmatic measurement tasks.

Furthermore, the data elucidated in Fig. 5c establish a direct correlation between the magnitude of the spectral shift and the degree of sequence complementarity between the probe and target ssDNA within the test solutions. Specifically, a higher sequence complementarity leads more pronounced spectral shifts, thereby indicating the sensor's acute sensitivity to the molecular binding events. In contrast, solutions characterized by wholly non-complementary ssDNA sequences exhibited negligible spectral shifts, affirming the sensor's specificity. The minor signal discrepancies noted in such instances are attributed to refractive index modifications spurred by ambient temperature variances.

These observations highlight the exceptional temperature stability of the fiber-optic DNA biosensor, a critical attribute that significantly augments its applicability in practical settings. The demonstrated stability, coupled with the sensor's specificity, vigorously validates the utility of this innovative biosensor across a broad spectrum of detection scenarios, underscoring its value in the flourishing field of biosensing technology.

Table 2 offers an in-depth comparison of the fiber-optic FP sensor explored in this study with existing fiber-optic DNA sensors documented in the literature. Notably, the FP sensor exhibits moderate refractive index sensitivity. Its standout feature, however, is the temperature sensitivity, recorded at just 0.15 nm °C^−1^. This value is significantly lower than what is typically optical fiber DNA biosensors, underscoring the sensor's advantageous low temperature sensitivity for practical applications where minimal thermal fluctuation is critical for accurate measurements. Moreover, its simplified design enhances the sensor's reliability and simplifies the demodulation process of the detection signal, rendering the sensor not only cost-effective but also user-friendly for practical applications.

**Table 2:** Performance comparison of fiber-optic DNA sensors

Sensor	Concentration (μM)	Temperature sensitivity (nm °C^−1^)	RI sensitivity (nm per RIU)	Wavelength shift (nm)
Tapered optical fiber^27^	0.001	—	1905.7	1.9
Dual-peak long period gratings^28^	0.5	—	794	1.044
Microfiber Bragg grating^29^	1	—	425	0.14
Micro-capillary-based evanescent^33^	5	—	628.975	0.054
Double microcavities FP sensor^34^	1	0.39	7122.63	1.22
Our work	1	0.15	1102.89	1.27

## 4. Conclusions

This study introduces a miniaturized fiber-optic Fabry–Perot biosensor that enables label-free, rapid, and reusable DNA diagnostics. The biosensor exhibits high specificity and sensitivity, accurately distinguishing ssDNA sequences with subtle differences, such as those with 2 and 7 mismatches. It achieves quick detection through primary wavelength shifts during DNA hybridization, demonstrating its potential for DNA diagnostics.

While the biosensor demonstrates considerable potential, its ability to detect in complex biological matrices beyond 1 μM DNA solutions has yet to be explored, highlighting a pivotal direction for forthcoming research. Subsequent efforts will aim at refining the sensor's design and broadening its diagnostic scope to include a more extensive array of DNA sequences and concentrations.

## Author contributions

Xin Shi: conceptualization, methodology, validation, writing – original draft. Yanhong Ma: data curation, resources. Yipeng Liao: software, formal analysis. Hoi Lut Ho: project administration, writing – review & editing.

## Conflicts of interest

There are no conflicts to declare.

## Supplementary Material
